# The association of parathyroid hormone with serum 25-hydroxyvitamin during pregnancy

**DOI:** 10.1017/jns.2022.110

**Published:** 2023-01-06

**Authors:** Elham Kazemian, Elham Madreseh, Fereidoun Azizi, Sepideh Ashrafivand, Soraya Saleh Gargari, Mohammad Ali Mansournia, Carol L. Wagner, Atieh Amouzegar

**Affiliations:** 1 Non-communicable Diseases Research Center, Alborz University of Medical Sciences, Karaj, Iran; 2 Endocrine Research Center, Research Institute for Endocrine Sciences, Shahid Beheshti University of Medical Sciences, Tehran, Iran; 3 Department of Epidemiology and Biostatistics, School of Public Health, Tehran University of Medical Sciences, Tehran, Iran; 4 Department of Gynecology & Obstetrics, Shohada Tajrish Educational Hospital, Shahid Beheshti University of Medical Sciences, Tehran, Iran; 5 Department of Pediatrics, Shawn Jenkins Children's Hospital, Medical University of South Carolina, Charleston, SC, USA

**Keywords:** Endocrine regulation, Pregnancy, PTH, Vitamins

## Abstract

It is currently debated whether vitamin D requirements during pregnancy differ from those during non-gravid states. In current analyses, we aimed to determine the best model for the association between PTH and serum 25-hydroxyvitamin D (25(OH)D) and the threshold for circulating 25(OH)D at which serum parathyroid hormone (PTH) is suppressed. This multicenter prospective cross-sectional study was conducted on 227 Iranian pregnant women aged 15–45 years in their third trimester of pregnancy. The locally weighted smoothing scatter plot (LOWESS) was used to determine the curvilinear shape of the 25(OH)D/PTH relationship. Linear and non-linear methods were employed to determine the best fit and cut-point for serum 25(OH)D concentration. The median serum 25(OH)D and corresponding serum PTH concentration were 17⋅26 (13⋅44–23⋅08) ng/ml and 19⋅46 (15⋅08–25⋅04) pg/ml in our study population, respectively. The LOWESS curve suggested a non-linear and monotonic with a negative slope relation between PTH (pg/ml) and serum 25(OH)D (ng/ml). The optimal model for the association between PTH and serum 25(OH)D was a one-term fractional polynomial (FP1) (AIC = 1640⋅463). The FP1 analysis identified the 25(OH)D threshold of 12⋅48 ng/ml at which serum PTH rapidly rose. The expected degree of PTH stimulation seems to have a linear trend as 25(OH)D falls below 40 ng/ml. 25(OH)D (ng/ml) and PTH (pg/ml) had a non-linear and monotonic relationship with a negative slope. Our data suggest that a 25(OH)D threshold of 12⋅48 ng/ml is sufficient for parathyroid hormone suppression, which could be used to screen for deficient individuals.

## Introduction

Pregnancy is a critical period of life during which a balanced intake of micronutrients and macronutrients is necessary for the prevention of maternal malnutrition and normal fetal growth and development^(1)^. Pregnant mothers experience significant anatomical and physiological changes to nurture and accommodate the developing fetus, altering their nutrient requirements^(2)^. In spite of the widespread use of prenatal vitamins, maternal vitamin D deficiency is common during pregnancy^(3)^. Vitamin D deficiency is reported to be associated with different adverse health outcomes in mothers, e.g. pre-eclampsia and preterm delivery^(4,5)^, and in their offspring, such as neonatal hypocalcemia, impaired skeletal development, respiratory infections, allergic diseases, obesity and type 1 diabetes during the early years of life^(3,6–9)^. In pregnancy, vitamin D requirements are likely greater than in a non-pregnant state^(10,11)^. This raises questions about the threshold to be considered sufficient for vitamin D status during pregnancy or the range for normal vitamin D function. An important part of this debate relies on inconsistencies regarding which indicators should be considered to determine maternal vitamin D status.

Biological interaction between circulating vitamin D and its upstream regulator, serum parathyroid hormone (PTH), has been proposed in view of uncertainty about outcomes^(12)^. As vitamin D is responsible for calcium absorption through diet, even mild vitamin D deficiencies can be compensated to maintain normal calcium availability^(13)^. Therefore, considering the classic endocrine function of vitamin D and its effects on calcium and bone metabolism, the biological interaction between serum 25-hydroxyvitamin D (25(OH)D) and PTH is commonly used to define the normal range of circulating 25(OH)D for calcium and bone metabolism^(12,13)^. In particular, the serum 25(OH)D status is determined by the point at which serum PTH begins to rise or is suppressed^(13,14)^. One question is to evaluate how circulating 25(OH)D correlates with PTH in pregnancy. To our knowledge, only a few studies investigated the association between 25(OH)D and PTH among pregnant women^(12,15,16)^. Based on a cohort of pregnant women, the threshold level of 25(OH)D needed to suppress PTH in pregnancy was similar to that found in postpartum women, despite the different shape of this relationship^(12)^. Another cross-sectional study by Haddow *et al.* indicated a weaker association of PTH/25(OH)D in pregnant women than in non-pregnant individuals^(15)^. Furthermore, since vitamin D is optimal for PTH suppression and bone and mineral metabolism varies among ethnic groups^(17,18)^, such investigations should ideally be conducted in different ethnic groups and regions. Therefore, in this cross-sectional study, we aimed to assess the PTH/25(OH)D relation and the optimal 25(OH)D concentration at which PTH is suppressed in a subgroup of Iranian pregnant women for the first time.

## Material and methods

### Study subjects

This was a multicenter prospective, cross-sectional study conducted on 227 pregnant women aged 15–45 years. We recruited pregnant women in the third trimester for the present study due to the greater importance of vitamin D status for fetal bone growth and optimal maternal and fetal health during this time^(19)^. In addition, infant cord 25(OH)D concentration is most strongly correlated with maternal status towards the end of the third trimester and had almost no correlation with maternal status before 20 weeks gestation^(20)^. Moreover, a weaker association of PTH/25(OH)D in the first trimester may make it an unreliable determinant for estimating vitamin D deficiency^(15)^. Pregnant women who attended antenatal care services and were willing to participate in the present study were enrolled between March 2013 and March 2014. All hospitals with obstetrics and gynecology wards in Tehran were stratified based on their socioeconomic status and location. A stratified systematic sampling method was used to select a centre that best represented the location and socioeconomic characteristics of each stratum, including Mostafa Khomeini (Centre of Tehran), Mahdieh (South of Tehran), Shohadaye Tajrish (North of Tehran), Bahman and Arash (West and East of Tehran). Participants from each stratum were selected based on the proportion of hospital admissions. Exclusion criteria were as follows: (1) history of parathyroid or adrenal gland disorders, granulomatous disease, renal dysfunction, hypoalbuminemia, thyrotoxicosis, sepsis, chemotherapy and blood transfusion; (2) the use of magnesium sulphate, lithium or thiazide medications; and (3) vitamin A intake ≥3000 μg/d and vitamin D ≥4000 IU/d. The final analysis was carried out in 227 women after excluding subjects who had abnormal PTH (<9 pg/ml) and calcium levels (≥10⋅5 mg/dl) (*n* 6) and individuals with missing values (*n* 1).

### Study measurements

Information regarding age, education, socioeconomic status, history of diseases, parity, gravidity, calcium and vitamin D intake from food and supplement and multivitamin consumption and dosage during pregnancy, exposure to sunlight^(21)^ and physical activity^(22)^ during pregnancy were obtained through an interview-administered questionnaire.

### Anthropometric measurement

Anthropometric measurements, including pregnancy height and weight, were measured at recruitment. Height was measured using the Secastadiometer without footwear and head facing forward. Body weight was determined to the nearest 0⋅1 kg, without shoes in light indoor clothing, using a portable digital scale. Body mass index (BMI) was calculated by dividing weight in kilogram by height in metres squared. Weight gain during pregnancy was determined by subtracting pre-pregnancy weight from the weight measured at study recruitment.

### Physical activity assessment

Physical activity during the last month was assessed by the questionnaire comprising nine questions regarding time spent in sitting position, walking, regular daily and sports activities besides six questions about physical demands of their job as well as light and heavy lifting on a regular working day. Total physical activity was reported as Metabolic Equivalent Task per kilogram per hour (kcal/kg/h)^(23)^.

### Sun exposure assessment

Sun exposure was assessed by a questionnaire including questions regarding hours of outdoor activities and body surface area exposed to sunlight while outdoors, sunscreen use and type of Islamic clothing.

### Laboratory measurements

A fasting venous blood sample was collected from each participant at recruitment in the third trimester of pregnancy. All laboratory measurements were carried out at the Research Institute for Endocrine Science (RIES) laboratory. Serum calcium and phosphorous were measured by photometric methods. Intact PTH and 25(OH)D concentrations were determined by the electrochemiluminescence immunoassay (ECLIA) method, using Roche Diagnostics kits and the Roche/Hitachi Cobas e-411 analyzer (Roche Diagnostics, GmbH, Mannheim, Germany). All intra- and inter-assay CVs were <2⋅6 for PTH and <7⋅5 for 25(OH)D concentrations.

### Statistical analyses

The Shapiro–Wilk's test was used to determine whether variables were normally distributed in this cohort of Iranian women. The preliminary analyses were conducted using a locally weighted smoothing scatter plot (LOWESS) to determine the curvilinear shape of the relationship. Also, linear and non-linear methods (i.e. univariate fractional polynomial (FP), regression spline and segmented regression) were used to determine the best fit and cut-point for serum 25(OH)D concentration. The cut-off values for circulating 25(OH)D were determined as the circulating 25(OH)D levels at which serum PTH is maximally suppressed or rapidly raised. The model with the lowest Akaike Information Criteria (AIC) value was considered the best fit.

In the secondary analyses, the 25(OH)D-specific PTH reference intervals and calcium-specific PTH percentiles were estimated using the normal-based methodology described by Royston and Wright^(24)^. This parametric approach is an extension of normal distribution considering skewness and kurtosis and is a robust methodology with efficient estimates.

Since the PTH had a non-Gaussian distribution, the modulus exponential-normal (MEN) 4-parameter model was used to provide a much more improved fit. A normal plot of the *Z*-scores was used to determine the goodness of fit of the MEN model.

In addition, we determined the threshold of circulating 25(OH)D based on the development of a composite of adverse pregnancy outcomes, including pre-eclampsia, pregnancy hypertension, gestational diabetes mellitus, severe nausea and vomiting during pregnancy, premature rupture of membranes (PROM) and premature delivery. Study participants were divided whether they developed adverse maternal outcomes. *χ*^2^ and independent *t* tests were first used to compare the serum vitamin D status of groups. In addition, we performed logistic regression analysis to evaluate the association between mothers’ vitamin D status and developing an adverse event during pregnancy after adjustment for potential confounders, including age, sun exposure, anthropometric measurement and physical activity. Finally, a receiver operator characteristic (ROC) curve was employed to determine the threshold and diagnostic ability of serum 25(OH)D concentrations to develop adverse pregnancy outcomes. However, this was an exploratory analysis, and considering adverse pregnancy events as a composite outcome for determining vitamin D outcome was not our primary goal. All statistical analyses were performed using Stata version 13.0 and MedCalc.

## Results

The baseline characteristics of the study participants are shown in Table 1. The study population comprised 227 pregnant women with median maternal and gestational age of 28 (26–31) years and 39 (38–39) weeks, respectively. The median serum 25(OH)D concentration was 17⋅26 (13⋅44–23⋅08) ranged from 4⋅33 to 50⋅88 ng/ml, and the corresponding serum intact PTH level was 19⋅46 (15⋅08–25⋅04) pg/ml. Considering the cut-off value of circulating 25(OH)D ≤12⋅48 ng/ml as vitamin D deficiency based on the best fit model for PTH/25(OH)D relation in our data, 45 (20 %) women were considered to be vitamin D-deficient. Whereas, according to available guidelines that suggest a 25(OH)D level <20 ng/ml for vitamin D deficiency^(25)^, 70 % (159) of the study participants had vitamin D deficiency. We noted higher circulating 25(OH)D concentration in women exposed to sunlight ≥15 compared with <15 min/d (17⋅69 ± 6⋅88 *v*. 22⋅1 ± 6⋅93 ng/ml, *P*-value 0⋅001) and 203 (89 %) of study participants had sun exposure > 15 min/d. Forty-four (18 %) subjects regularly used calcium-vitamin D supplements, and 144 (63 %) took a multivitamin supplement.

**Table 1.** Baseline characteristics of study participants

Characteristics	(*N* 227)
*Demographic and physiologic characteristics*	
Maternal age (years)	28 (26–31)
Gestational age (weeks)	39 (38–39)
Education, *N* (%)	
Less than high school	16 (7 %)
Diploma	117 (52 %)
Academic	94 (41 %)
Deliveries, *N* (%)	
0	136 (60 %)
1	75 (33 %)
≥2	16 (7 %)
Pregnancy, *N* (%)	
1	120 (53 %)
2	86 (38 %)
≥3	21 (9 %)
*Physical activity*	
MET (MET-min/wk)	24 (22–28)
*Sun exposure*	
Mean sun exposure, *N* (%)	
<15 min/d	202 (89 %)
≥15 min/d	25 (11 %)
Sunscreen use, *N* (%)	179 (79 %)
*Body composition*	
Weight (kg)	66 (60–70)
BMI (kg/m^2^)	25 (23–27)
Gestational weight gain (kg)	12 (10–13)
*Laboratory measurements*	
Intact PTH (pg/ml)	19⋅46 (15⋅08–25⋅04)
25(OH)D (ng/ml)	17⋅20 (13⋅42–23⋅04)
Calcium (mg/dl)	9⋅61 (9⋅32–9⋅90)
Phosphorous (mg/dl)	3⋅59 (3⋅08–4⋅07)

MET, Metabolic equivalent of task; PTH, Parathyroid hormone; 25(OH)D, 25-hydroxyvitamin D.

Values are presented as median (Q1, Q3) and categorical variables are presents as *n* (% per row).

**Fig. 1.**
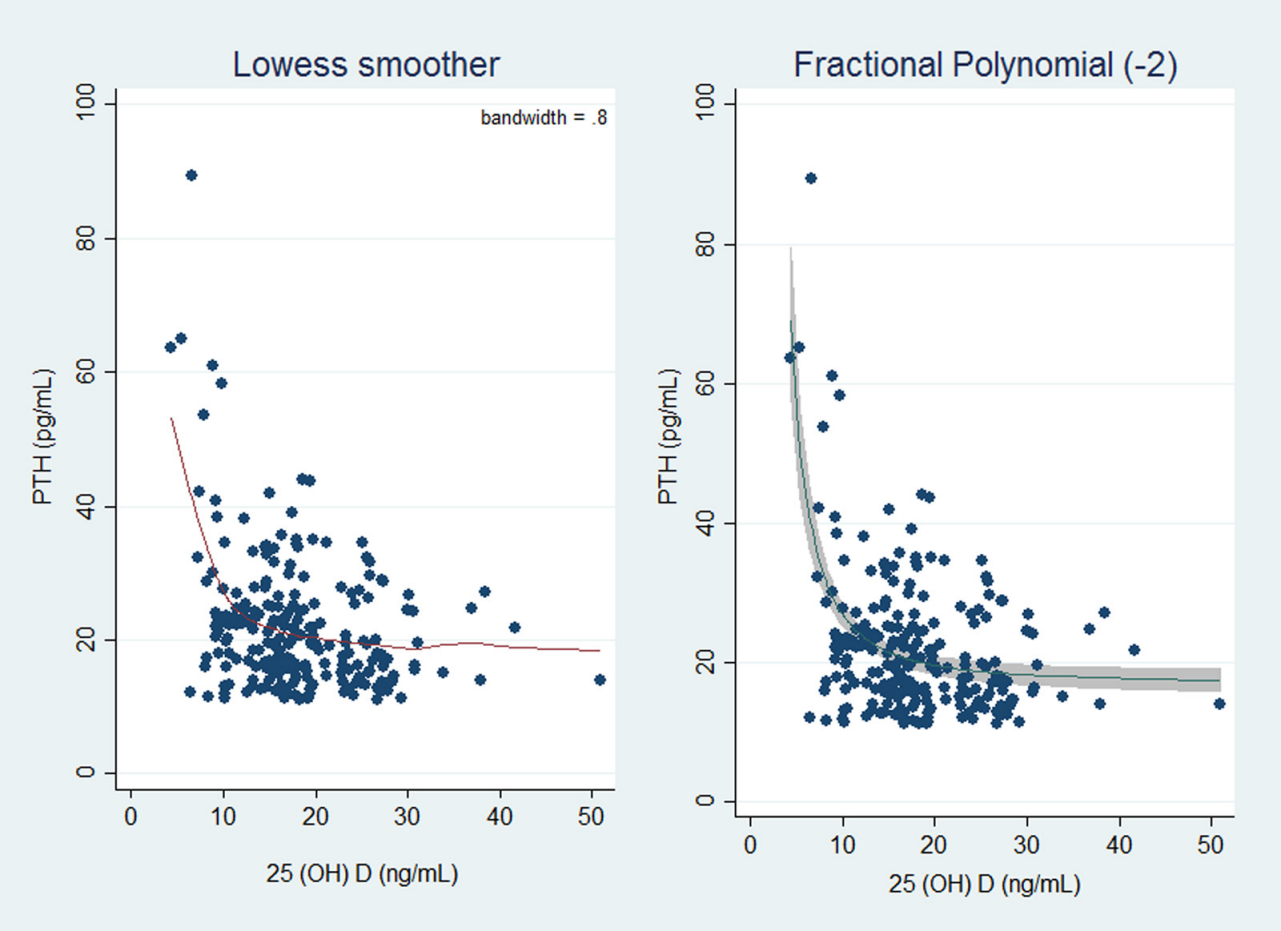
Plot of original data with the locally weighted smoothing scatter plot (LOWESS) model curve (a) and fractional polynomial with power = −2 (b). PTH, parathyroid hormone; 25(OH)D, 25-hydroxyvitamin D.

Spearman correlation showed an inverse relationship between serum 25(OH)D concentration and PTH (Spearman's *r* = −0⋅233, *P*-value < 0⋅001). The LOWESS curve also suggested a non-linear and monotonic with a negative slope relation between PTH and serum 25(OH)D (Fig. 1).

The cut-off value of vitamin D deficiency was defined as the circulating 25(OH)D levels at which serum PTH is maximally suppressed or rapidly raised. Therefore, segmented regression and FP analyses were employed to estimate the best model for the association between PTH and serum 25(OH)D and the circulating 25(OH)D threshold. The non-nested model selection was performed based on the minimum Akaike Information Criterion (AIC) as the criteria for the goodness of fit and model complexity. Six segmented regression models were constructed as follows: (1) Linear–linear model (Threshold = 10⋅34, AIC = 1748⋅365); (2) Exponential–linear model (Threshold = 10⋅33, AIC = 1748⋅369); (3) Linear mode model–plateau (Threshold = 10⋅48, AIC = 1748⋅612); (4) Exponential model–plateau (Threshold = 10⋅47, AIC = 1748⋅612); (5) Quadratic–linear model (Threshold = 10⋅34, AIC = 1750⋅365) and (6) Quadratic model–plateau model (Threshold = 10⋅48, AIC = 1750⋅611).

In FP models, for both one-term FP1 and two-term FP2 models, the best possible choice of powers was −2 in FP1. This selection was based on likelihood ratio tests. The fitted FP model was:
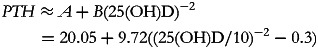
Where the estimated effects (and standard error) were: A = 20⋅05 (0⋅63), B = 9⋅72 (1⋅11) and the unit for 25(OH)D was ng/ml. For this model, the cut-point (a point in the PTH-25(OH)D curve at which PTH is maximally suppressed) was approximated by calculating the point at which the change (Δ) in PTH per unit change in 25(OH)D was approximately zero. ΔPTH ≈ 0 when Δ PTH was considered 1/0000th of the sd for PTH, i.e. 10⋅42/100 000 ≈ 0⋅001 pg/ml. This approach indicated the estimated cut-point of 12⋅48 ng/ml (|( − 2B/10Δ)^1/3^|).

Our findings showed that the optimal model was one-term FP1 with the 25(OH)D threshold of 12⋅48 ng/ml (AIC = 1640⋅463) (Table 2).

**Table 2.** Models for the association between 25(OH)D and PTH and estimated 25(OH)D threshold at which PTH stimulates to increase

Models	25(OH)D threshold (ng/ml)	AIC
FP1	12·48	1640·463
Segmented regression		
Linear–linear model	10·34	1748·365
Exponential–linear model	10·33	1748·369
Linear mode model–plateau	10·48	1748·612
Exponential model–plateau	10·47	1748·612
Quadratic–linear model	10·34	1750·365
Quadratic model–plateau	10·48	1750·611

FP1, One-term fractional polynomial; 25(OH)D, 25-hydroxyvitamin D; AIC, Akaike's information criterion.

**Fig. 2.**
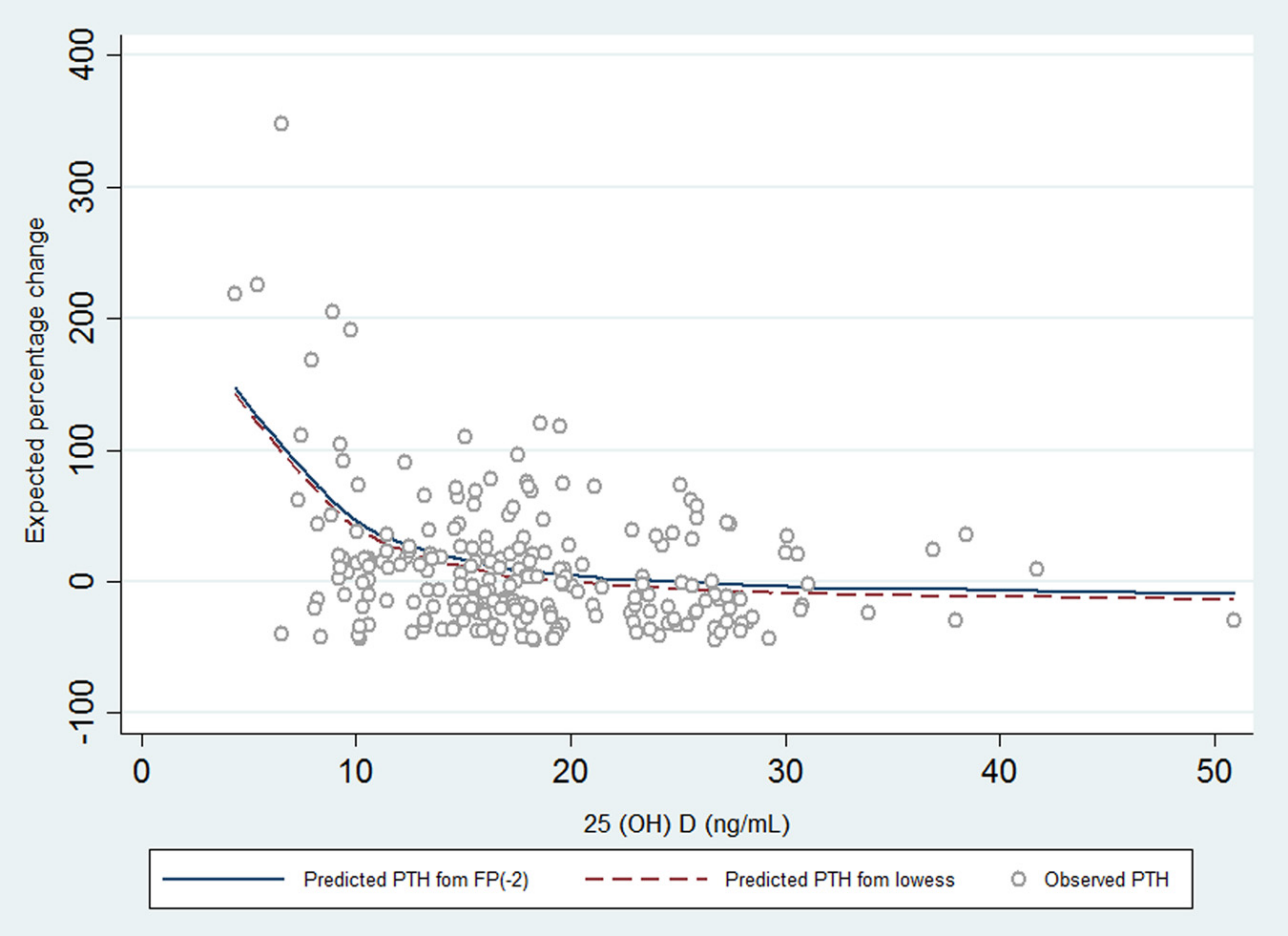
Degree of PTH stimulation at different levels of 25(OH)D, shown as the expected percentage change in serum PTH compared with PTH when 25(OH)D is 40 ng/ml during third trimester of pregnancy. PTH, parathyroid hormone; 25(OH)D, 25-hydroxyvitamin D.

The expected percentage change in serum PTH compared with PTH when 25(OH)D = 40 ng/ml is depicted in Fig. 2. The degree of PTH stimulation declines as the concentration of 25(OH)D rises until it reaches an optimal level (10, 12 ng/ml), and then, it gradually approaches zero. For example, a 40 or 20 % increase in serum PTH was expected at 25(OH)D concentration of 10 and 12 ng/ml, respectively, compared with the PTH level at 25(OH)D concentration of 40 ng/ml.

Table 3 shows the estimated 25(OH)D and calcium categories-specific PTH values for 2·5th, 5th, 10th, 25th, 50th, 75th, 90th, 95th and 97·5th centiles (95 % confidence interval) based on non-parametric models. Using the 2·5th and the 97·5th percentiles for calculating reference intervals in the 25(OH)D <10 ng/ml, 10 < 25(OH)D <20 ng/ml, 20 < 25(OH)D <30 ng/ml and 25(OH)D >30 ng/ml categories, resulted in (11·5–89·45), (11·25–40·71), (11·20–34·57) and (13·93–27·12) pg/ml for PTH.

**Table 3.** Observed PTH centile and 95 % CI based on 25(OH)D and calcium categories

	Observed PTH centile (95 % CI*) (pg/ml)								
	2·5th	5th	10th	25th	50th	75th	90th	95th	97·5th
Total (*n* 227)	11·29 (11·13, 11·65)	11·67 (11·29, 12·26)	12·57 (12·05, 13·27)	15·08 (13·93, 15·87)	19·46 (17·88, 20·72)	25·04 (23·90, 27·44)	33·92 (30·69, 36·30)	40·14 (34·59, 54·30)	55·02 (40·42, 64·97)
25(OH)D categories (ng/ml)									
25(OH)D <10 (*n* 22)	11·5 (11·50, 15·52)	11·58 (11·50, 17·20)	13·20 (11·5, 19·99)	19·75 (12·90, 25·05)	29·39 (21·34, 42·90)	54·79 (31·80, 64·74)	64·63 (54·34, 89·45)	85·79 (61·06, 89·45)	89·45 (63·76, 89·45)
10 < 25(OH)D <20 (*n* 136)	11·25 (11·12, 11·60)	11·38 (11·21, 12·36)	12·47 (11·52, 13·21)	15·54 (13·52, 16·24)	20·06 (18·02, 22·01)	24·64 (23·40, 27·10)	33·68 (28·46, 35·01)	35·16 (33·82, 42·36)	40·71 (34·97, 44·02)
20 < 25(OH)D <30 (*n* 57)	11·20 (11·10, 12·12)	11·71 (11·10, 12·58)	12·34 (11·39, 13·55)	13·79 (13·06, 15·08)	16·90 (15·08, 18·70)	21·58 (18·79, 27·58)	29·02 (26·34, 34·16)	32·50 (28·70, 34·64)	34·57 (30·15, 34·64)
25(OH)D >30 (*n* 12)	13·93 (13·93, 14·31)	13·93 (13·93, 15·00)	13·94 (13·93, 15·59)	15·19 (13·93, 21·16)	20·62 (15·12, 24·69)	24·64 (20·08, 27·12)	27·01 (24·38, 27·12)	27·12 (24·84, 27·12)	27·12 (26·12, 27·12)
Calcium categories (mg/dl)									
Calcium <9·32 (*n* 57)	11·11 (11·10, 11·36)	11·20 (11·09, 12·05)	11·75 (11·13, 13·41)	15·48 (12·54, 16·89)	19·42 (16·90, 21·80)	23·84 (21·82, 32·98)	35·68 (27·63, 43·44)	42·26 (34·96, 65·05)	55·43 (39·10, 65·05)
9·32 < Calcium <9·61 (*n* 58)	11·29 (11·21, 12·10)	11·61 (11·21, 12·71)	12·25 (11·42, 13·81)	14·66 (13·23, 16·23)	18·87 (16·23, 22·52)	24·66 (22·64, 28·85)	32·83 (26·57, 34·63)	34·57 (31·59, 58·28)	47·05 (34·01, 58·28)
9·61 < Calcium <9·89 (*n* 55)	11·6 (11·50, 13·01)	12·15 (11·5, 13·60)	13·34 (11·78, 14·46)	15·49 (13·75, 17·25)	19·69 (17·24, 22·71)	27 (22·63, 34·07)	39·80 (30·49, 60·52)	55·09 (36·12, 89·45)	78·06 (41·95, 89·45)
Calcium >9·89 (*n* 57)	11·29 (11·29, 12·54)	12·03 (11·29, 12·74)	12·68 (11·41, 13·92)	14·61 (13·15, 16·05)	19·62 (16·07, 23·34)	25·75 (23·50, 28·36)	31·54 (27·75, 37·76)	34·49 (30·00, 63·63)	52·27 (33·27, 63·63)

* Binomial method was used for calculating confidence intervals.

PTH, parathyroid hormone; 25(OH)D, 25-hydroxyvitamin D; CI, confidence interval.

Using the 2·5th and the 97·5th percentiles for calculating reference intervals in the calcium <9·32 mg/dl, 9·32 < calcium <9·61 mg/dl, 9·61 < calcium <9·89 mg/dl and calcium >9·89 mg/dl quantiles resulted in (11·11–55·43), (11·29–47·05), (11·6–78·06) and (11·29–52·27) pg/ml for PTH.

**Fig. 3.**
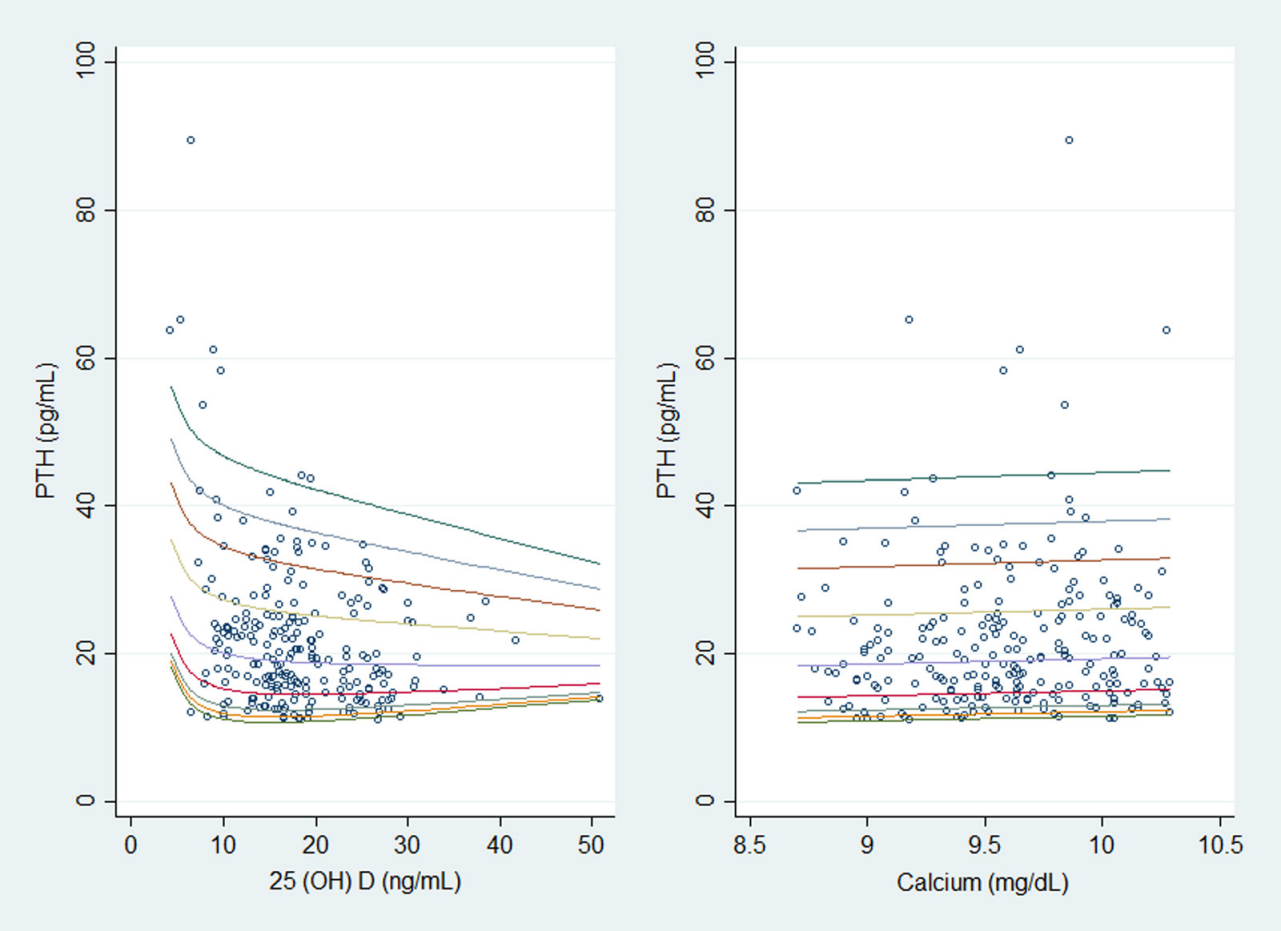
Trend of PTH percentiles (2·5th, 5th, 10th, 25th, 50th, 75th, 90th, 95th and 97·5th) in pregnant women as a function of the 25(OH)D (a) and calcium (b), resulted from modulus exponential-normal (MEN) models. PTH, parathyroid hormone; 25(OH)D, 25-hydroxyvitamin D.

Corresponding PTH percentiles for each level of 25(OH)D and calcium based on MEN models are plotted in Fig. 3. The 2·5th and 97·5th percentile values of PTH decreased significantly with increasing circulating 25(OH)D concentrations (Fig. 3(a)), while did not fluctuate with increasing calcium (Fig. 3(b)).

**Fig. 4.**
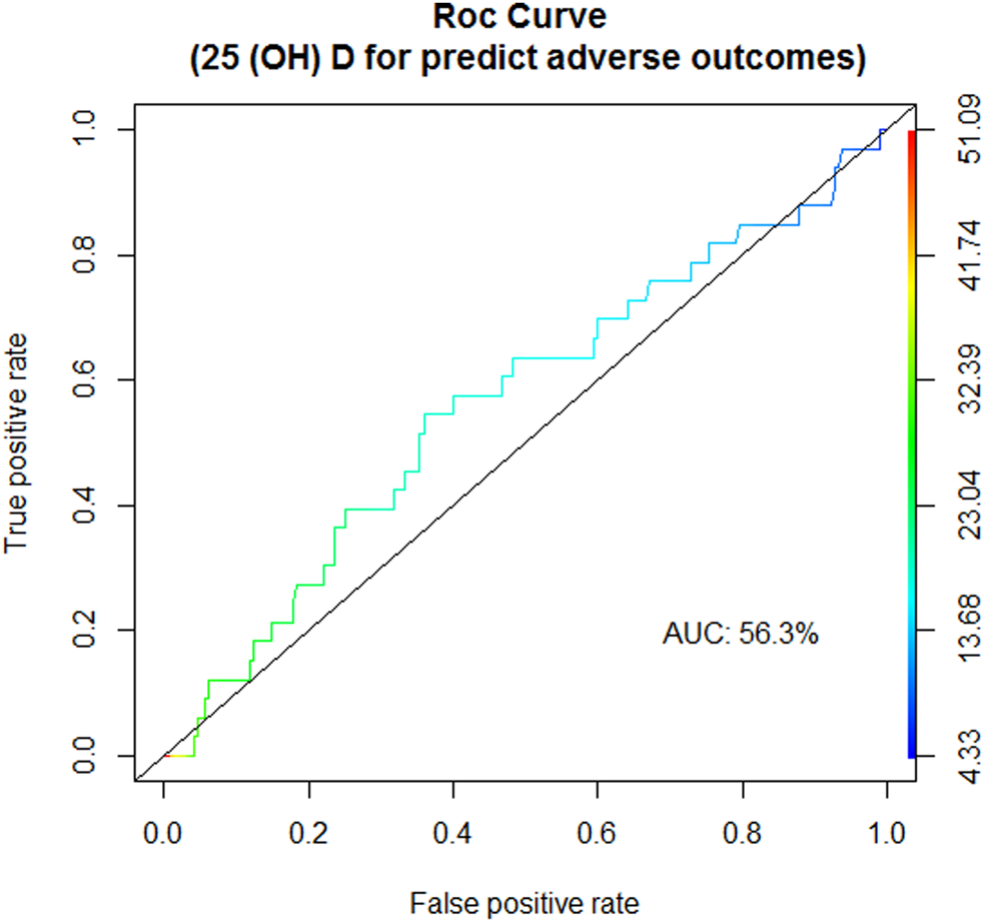
The receiver operating characteristic (ROC) curves for circulating 25-hydroxyvitamin D concentration to discriminate between maternal vitamin D sufficiency and deficiency.

Our findings showed no significant association between circulating 25(OH)D concentration and development of composite adverse outcomes during pregnancy either in an unadjusted analysis or after adjustment for age, sun exposure, anthropometric measurement and physical activity (results are not shown). The ROC curve analysis to determine the threshold of serum 25(OH)D concentrations for developing adverse pregnancy outcomes identified the value of 18·45 ng/ml (Fig. 4). However, the area under the curve (AUC) for the ROC curve plot in current data was 0·563, indicating that the occurrence of adverse outcomes during pregnancy was not properly predicted by vitamin D status in our study population.

## Discussion

The LOWESS curve suggested a non-linear and monotonic with a negative slope relation between PTH and serum 25(OH)D. The segmented regression and FP analyses were employed to determine the best model for the association between serum PTH and 25(OH)D concentrations. According to our results, the best model to predict endocrine vitamin D function in pregnancy was FP1 with a 25(OH)D threshold of 12·48 ng/ml at which serum PTH rapidly rose. We observed approximately a linear trend between serum PTH and 25(OH)D when serum 25(OH)D falls below 40 ng/ml. In a study by Hysaj *et al.*, 25(OH)D level of 18·9 ng/ml was suggested as an inflection point for the maximal suppression of PTH among pregnant Swiss women^(16)^. It appears ethnicity and resident region may affect the threshold for vitamin D deficiency among pregnant women. In another study in Iranian adolescents aged 10–16 years, the cut-off points for mild, moderate, and severe vitamin D deficiency based on 25(OH)D/PTH relation was 33, 21 and 13 ng/ml, respectively, which was comparable to 12·48 ng/ml in our analysis^(26)^. However, other factors such as BMI, season and age assumed to affect this relation, should also be considered^(12,15,27,28)^.

The ROC curve analysis suggested the occurrence of composite adverse pregnancy outcomes was not a good discriminator for defining vitamin D deficiency in our study population. Clinical endpoint such as the history of falls and risk of fracture and chronic disease as well as assessment of the maximal suppression of PTH by 25(OH)D are suggested as indicators to evaluate the optimal serum 25(OH)D levels^(29)^. It should be kept in mind that vitamin D deficiency should be diagnosed before developing adverse health outcomes, as most individuals with vitamin D deficiency are unaware of their serum vitamin D levels. Therefore, the thresholds for vitamin D deficiency should be established by determining which outcomes could best predict deficiency, assuming to improve the health of individuals with low 25(OH)D levels. In our study, developing a composite of adverse outcomes during pregnancy was not a suitable indicator for determining the cut-point to discriminate between vitamin D deficiency and sufficiency. However, it should be mentioned that assessing adverse outcomes during pregnancy was not our primary goal. Owing to that, our small sample size may lead to insufficient power of the current investigation to detect the association between adverse pregnancy outcomes during pregnancy and 25(OH)D concentration. Yet, controversies continue concerning which outcomes should be considered to define optimal serum 25(OH)D levels.

During pregnancy, maternal intestinal calcium absorption is enhanced to provide calcium requirement for the developing fetus^(30)^. Correspondingly, the association between serum PTH and 25(OH)D during gestation may be distinct from the relation of the non-gravid state^(12,15,31)^. For example, a study by Haddow *et al.* indicated a relationship between PTH and 25(OH)D was weaker in early pregnancy than in non-pregnant adults^(15)^. However, the inverse association between PTH and 25(OH)D has been shown in other studies conducted in pregnant adults and adolescents^(12,16,32–34)^. In our population, the LOWESS curve documented a non-linear and monotonic with a negative slope relation between PTH and serum 25(OH)D, which was similar to curvilinear fits employed to model this relation among third-trimester pregnant women in a study by Hysaj *et al.*^(16)^. Furthermore, our findings demonstrated a linear trend in the degree of PTH stimulation as 25(OH)D falls below 40 ng/ml. Therefore, it is proposed that PTH secretion is elevated in response to 25(OH)D deficiency to meet the calcium demands of both the mother and the fetus^(33)^.

There is a clinical need to define a threshold 25(OH)D concentration below which serum PTH is no longer suppressed and considered an endocrine indicator of physiological vitamin D insufficiency. This cut-point in our data was determined on FP1 analysis with the 25(OH)D threshold of 12·48 ng/ml below which serum PTH rapidly rose, which is near to cut-points previously identified in non-pregnant adults^(29)^. Nevertheless, a study by Kramer *et al.*, conducted among Canadian women in their late pregnancy revealed a linear association between 25(OH)D and PTH up to a 25(OH)D threshold of 82 nmol/l, below which PTH was no longer suppressed^(12)^. Whereas, in a study by Haddow *et al.*, a weak PTH and 25(OH)D relationship suggested that threshold analysis is unreliable for determining vitamin D status during pregnancy^(15)^. Different vitamin D deficiency thresholds documented by studies may be explained by racial differences in PTH/25(OH)D relationship. For example, in a study by Okonofua *et al.*, lower concentrations of 25(OH)D in Asian women were accompanied by only a minimal increase in PTH levels compared with their Caucasian counterparts, likely due to the fact that low levels of 25(OH)D in Asian women could be partially compensated by higher synthesis of 1,25-dihydroxyvitamin D (1,25(OH)2 D)^(35)^. Additionally, it is possible that the pattern of relationship between 25(OH)D and PTH observed in our pregnant population was driven by the high prevalence of vitamin D deficiency among Tehranian residents^(36)^. A cross-sectional study by Haddow *et al.* noted an inverse association of PTH with 25(OH)D concentrations during early pregnancy among African American women when 25(OH)D <20 ng/ml^(15)^. It should also be noted that 25(OH)D was measured in the third trimester of pregnancy in our study while others measured it earlier in pregnancy, which may contribute to different association patterns of serum 25(OH)D and PTH levels observed in various studies. However, the underlying mechanism on how race or a lower 25(OH)D status may contribute to discrepancies between studies should be investigated in future studies.

This is the first study to assess the PTH/25(OH)D relation as well as optimal 25(OH)D concentration among a subgroup of Iranian pregnant women. In the current analyses, we applied rigorous statistical modelling to determine the best fit for the PTH/25(OH)D relation in a subgroup of pregnant women. One limitation of this study is its cross-sectional design with single time point measurement during the third trimester of pregnancy. We could not determine whether the observed association was a feature of the study population or gestation. In addition, we did not assess the relationship between serum 25(OH)D and PTH in early and mid-pregnancy, which may differ from late pregnancy. Furthermore, our study did not have enough power to detect the effect of vitamin D status on pregnancy comorbidities. Lastly, we did not measure the dietary intake of vitamin D and calcium. However, it should be noted that there are a few naturally occurring food sources of vitamin D and vitamin D is mostly obtained from dietary supplements in the Iranian diet^(37,38)^. However, sun exposure, which is considered to be the main source of vitamin D, was considered in the current analyses. Future longitudinal studies with a larger sample size initiated in the first trimester of pregnancy to provide comprehensive insight into non-endocrine aspects of vitamin D during pregnancy are suggested.

In brief, in a subgroup of Iranian women with significant and often profound vitamin D deficiency evaluated at the start of the third trimester, the shape of the relationship between 25(OH)D and PTH was non-linear and monotonic with a negative slope. Our findings showed that the optimal model of PTH/25(OH)D relation was a one-term FP1. According to FP1 analysis, the vitamin D deficiency cut-point for the classical endocrine function using serum PTH as a marker was the 25(OH)D threshold of 12·48 ng/ml, at which serum PTH quickly rose. These analyses suggest that low 25(OH)D may result in PTH synthesis, which increases bone resorption in pregnant women to meet maternal and fetal calcium requirements. These findings contribute to a growing body of evidence regarding calcitropic hormone responses during pregnancy. Establishing thresholds for vitamin D deficiency among this vulnerable group of individuals would allow them to undergo screening and identify deficient individuals early before developing adverse health outcomes associated with vitamin D deficiency.
